# Diffusion of Immunoglobulin G in Shed Vaginal Epithelial Cells and in Cell-Free Regions of Human Cervicovaginal Mucus

**DOI:** 10.1371/journal.pone.0158338

**Published:** 2016-06-30

**Authors:** Ying-Ying Wang, Holly A. Schroeder, Kenetta L. Nunn, Karen Woods, Deborah J. Anderson, Samuel K. Lai, Richard A. Cone

**Affiliations:** 1 Department of Biophysics, Johns Hopkins University, Baltimore, Maryland, United States of America; 2 Division of Molecular Pharmaceutics, Eshelman School of Pharmacy, University of North Carolina – Chapel Hill, Chapel Hill, North Carolina, United States of America; 3 UNC/NCSU Joint Department of Biomedical Engineering, University of North Carolina – Chapel Hill, Chapel Hill, North Carolina, United States of America; 4 Department of Microbiology & Immunology, University of North Carolina – Chapel Hill, Chapel Hill, North Carolina, United States of America; 5 Departments of Obstetrics/Gynecology, Microbiology and Medicine, Boston University School of Medicine, Boston, Massachusetts, United States of America; University of California, Merced, UNITED STATES

## Abstract

Human cervicovaginal mucus (CVM) is a viscoelastic gel containing a complex mixture of mucins, shed epithelial cells, microbes and macromolecules, such as antibodies, that together serve as the first line of defense against invading pathogens. Here, to investigate the affinity between IgG and different mucus constituents, we used Fluorescence Recovery After Photobleaching (FRAP) to measure the diffusion of IgG in fresh, minimally modified CVM. We found that CVM exhibits substantial spatial variations that necessitate careful selection of the regions in which to perform FRAP. In portions of CVM devoid of cells, FRAP measurements using different IgG antibodies and labeling methods consistently demonstrate that both exogenous and endogenous IgG undergo rapid diffusion, almost as fast as in saline, in good agreement with the rapid diffusion of IgG in mid-cycle endocervical mucus that is largely devoid of cells. This rapid diffusion indicates the interactions between secreted mucins and IgG must be very weak and transient. IgG also accumulated in cellular debris and shed epithelial cells that had become permeable to IgG, which may allow shed epithelial cells to serve as reservoirs of secreted IgG. Interestingly, in contrast to cell-free regions of CVM, the diffusion of cell-associated IgG was markedly slowed, suggesting greater affinity between IgG and cellular constituents. Our findings contribute to an improved understanding of the role of IgG in mucosal protection against infectious diseases, and may also provide a framework for using FRAP to study molecular interactions in mucus and other complex biological environments.

## Introduction

The human vaginal epithelium is coated with a layer of viscoelastic mucus gel comprised of long and heavily glycosylated mucin molecules that are cross-linked, entangled and bundled to form a porous network with large mesh spacings (average ~340 ± 70 nm [1]), much larger than antibodies such as IgG and most mammalian viruses. Mucus secretions in the vagina are derived primarily from mucin-secreting glands in the endocervical canal [2]. As cervical mucus enters the vagina, it is modified by fluid and ion exchange [3,4], secreted or transudated antibodies, shed epithelial cells and vaginal microbiota [5,6]. As a result, mucus overlaying the vaginal epithelium is frequently referred to as cervicovaginal mucus (CVM) to emphasize both its origin and the unique physicochemical properties that differentiate CVM from endo-cervical mucus.

CVM not only serves as a lubricant minimizing physical trauma during coitus, but also provides an unstirred adherent layer of viscoelastic gel that can slow or prevent pathogens in semen from directly contacting the vaginal epithelium. Although native, acidic CVM was previously shown capable of trapping viruses [1,7], this trapping potency is generally lost when the pH of CVM is neutralized, as typically occurs when exposed to semen [7–9]. We have recently shown that IgG that binds to the surface of Herpes Simplex Virus (HSV), including endogenous IgG present in CVM and also exogenously added IgG, can trap (immobilize) HSV virions in human CVM at sub-neutralizing concentrations in an Fc- and N-glycan dependent manner [9]. Furthermore, by trapping HSV virions in mucus, IgG directly protects against vaginal transmission of HSV in mice. This largely underappreciated mechanism of mucosal immune protection is presumably due to IgG-mucin bonds; however, there continues to be substantial debate over the nature of IgG-mucin bonds in native mucus secretions. Many investigators have hypothesized that the trapping effect is mediated by high-affinity bonds between antibodies and mucus components, as first proposed by Kremer and Jager [10] and more recently by others [11]. In contrast, our previous measurements of exogenous IgG in cervical mucus and CVM suggested IgG only forms low-affinity, transient bonds with mucins, which would in turn allow individual antibodies to diffuse rapidly through mucus and quickly accumulate on invading pathogens [9,12]. Here, we sought to reconcile these opposing views by performing careful and comprehensive diffusion measurements of both exogenous and endogenous IgG in CVM.

A major challenge to studying interactions between different mucus constituents, such as IgG molecules and mucins, is the highly viscoelastic nature of physiological mucus gels, which makes mucus incompatible with many common methods for quantifying binding rates or affinities, including surface plasmon resonance, biolayer interferometry and atomic force microscopy. Since mucus is a hydrogel whose properties can be markedly altered by dilution, performing studies with heavily diluted mucus or purified mucin solutions, which no longer behave as a viscoelastic gel, carries the risk of either altering the affinity of the interactions or possibly eliminating detectable interactions altogether. Fluorescence microscopy provides an experimental approach that enables studies without appreciable dilution of the mucus gel, and has been broadly applied to characterizing the barrier properties of mucus [1,13–18]. Fluorescence Recovery After Photobleaching (FRAP), in particular, is a powerful tool for quantifying the affinity of molecular interactions in mucus; indeed, FRAP was previously used in landmark studies demonstrating that many proteins, including IgG and even virus-like particles, can diffuse through human mid-cycle cervical mucus at nearly the same rates as they do in saline. Nevertheless, a major difference between CVM and cervical mucus is the presence of substantial quantities of shed epithelial cells and communities of microbes that alter the composition and other properties of CVM. Vaginal epithelial cells express cell-associated mucins (e.g. MUC1, MUC4, and possibly MUC16) [14,19], which have been suggested to exhibit high affinity interactions with IgG [20]. Importantly, shed cells also introduce significant spatial variations in FRAP measurements. Here, we show that interpretation of FRAP experiments in CVM depends critically on whether the regions of interest (ROI) for FRAP illumination pass through cells or cell-free regions within CVM, as well as on the choice of fluorescent labeling approach.

## Results & Discussion

### Spatial variations in the distribution of IgG in CVM

**Fig 1:**
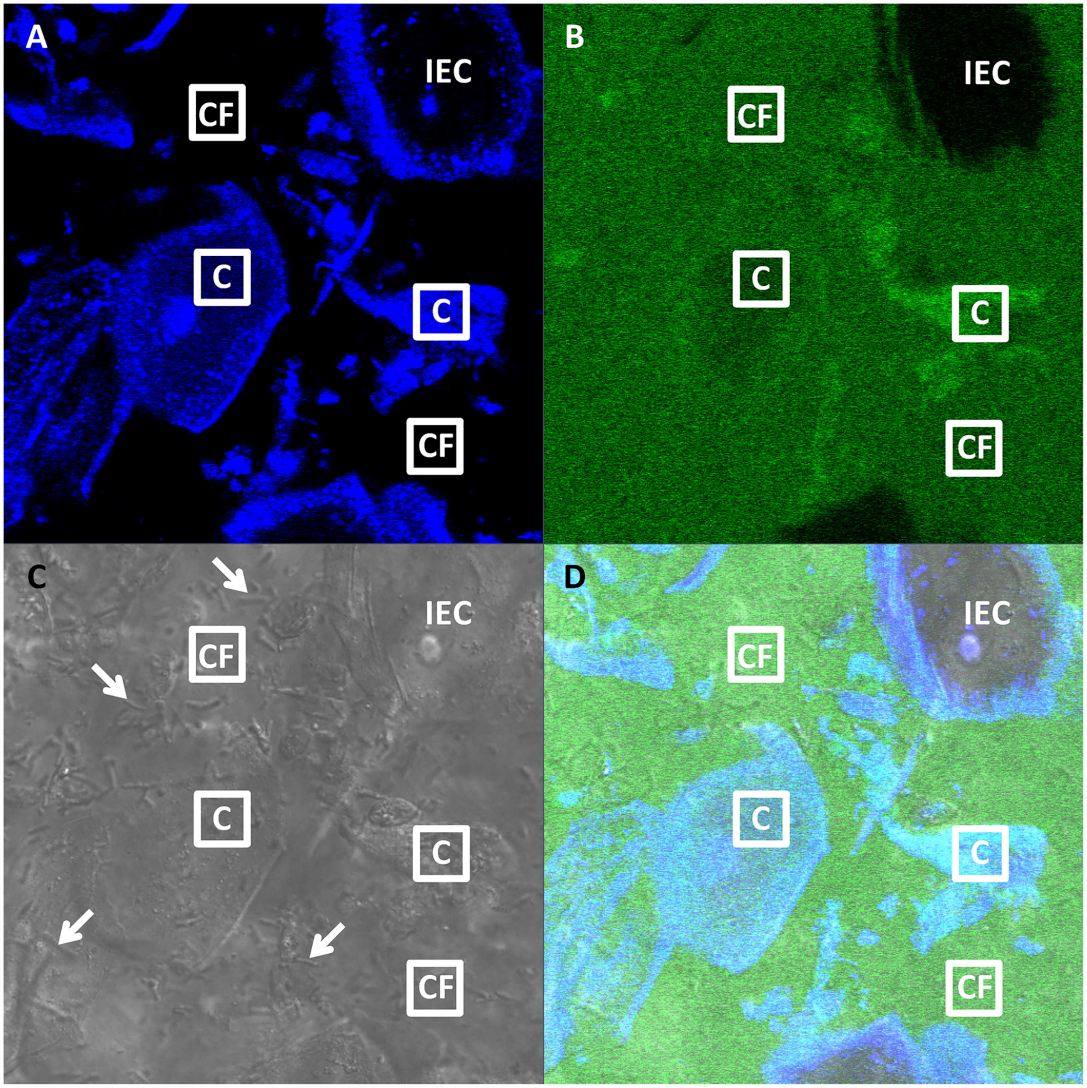
Representative confocal image of FITC-labeled human intravenous immunoglobulin (IVIG) in pH-neutralized human CVM. (A) Blue, (B) green, (C) DIC and (D) composite channel images, with sample “Cell-Free” (“CF”) and “Cell” (“C”) ROIs measuring approximately 10 μm x 10 μm indicated. An example of an intact epithelial cell (“IEC”) without absorbed antibody is also shown. Arrows indicate a subset of lactobacilli. Cells are identified from their morphology and blue auto-fluorescence. Intact epithelial cells do not absorb antibody and thus appear dark in the green channel. “Cell” ROIs are selected within cells that have absorbed antibody and thus appear green at comparable or greater intensity than the surrounding mucus. “Cell-Free” ROIs are selected outside of cells, where the green-fluorescent antibody is relatively uniformly distributed. For comparison, a representative confocal image of CVM without FITC-labeled antibody is presented in S1 Fig.

CVM contains a high density of largely dead, shed vaginal epithelial cells that are at varying stages of degradation. Based on pilot studies, we have measured the concentration of shed cells in CVM to be over 50 million cells/mL, which corresponds to ~10% of CVM volume occupied by cells. Cells are shed and retained in the vagina for different periods of time, depending on when and potentially where in the vagina they were shed. Mucus clearance from the vagina is estimated to occur over several hours [21,22], but may vary spatially within the vagina. At any given time, some cells in collected CVM may have been freshly shed from the epithelium and still exhibit intact cell membranes, while others may have been degrading over the course of a day or more and have highly permeabilized membranes. As shown in Fig 1, due to the auto-fluorescence of cells and cellular bodies, cells and cell structures can be readily identified simply by scanning the image plane using a UV laser. When we incubated fluorescently labeled IgG in CVM, we observed that some shed epithelial cells absorbed fluorescently labeled IgG to levels indistinguishable from or visually greater than cell-free regions, while other cells remained dark, presumably due to a still intact, IgG-impermeable cell membrane. Absorption of IgG by shed cells was even more striking in washed vaginal epithelial cells (S2 Fig). Since IgG is the predominant immunoglobulin in CVM (>90%) [9,23,24], it is likely that a substantial fraction of shed vaginal epithelial cells contain absorbed endogenous IgG at any given time. Thus, in our studies below, we paid careful attention to selecting ROI in either cell-free regions of CVM or within cells.

### Slower recovery kinetics of IgG in cellular vs. cell-free regions of CVM

**Fig 2:**
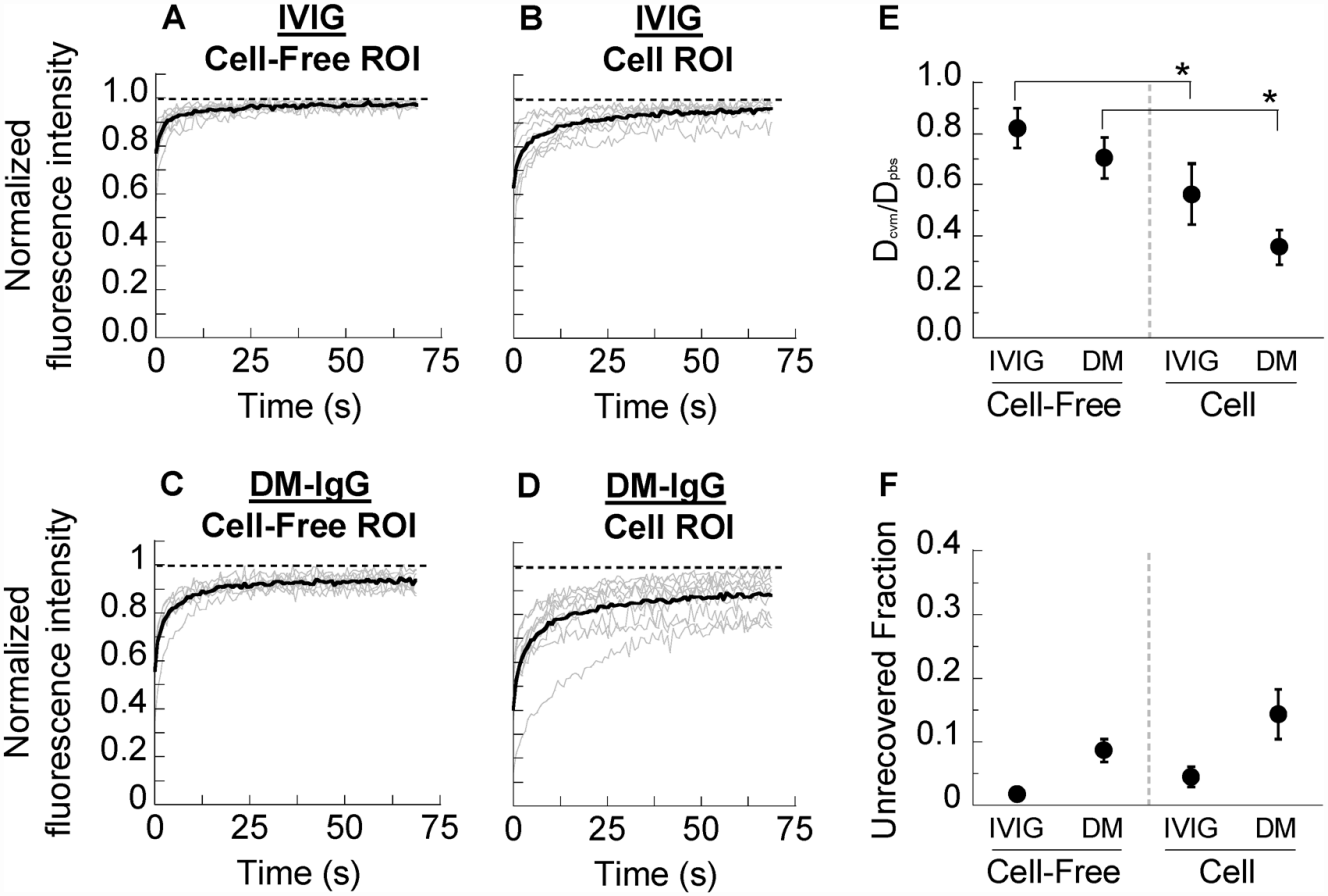
FRAP analysis of human intravenous immunoglobulin (“IVIG”) and donor-matched purified IgG (“DM-IgG” or “DM”) in pH-neutralized human CVM. Normalized fluorescence intensities over time for photobleached ROIs of FITC-labeled (A,B) IVIG and (C,D) DM-IgG in human CVM. ROIs are distinguished based on whether they are (A,C) in cell-free regions of the sample (“Cell-Free ROI”) or (B,D) within cells (“Cell ROI”). Thin grey lines represent individual measurements of distinct ROIs, while thick black lines represent the average. Dashed lines represent a normalized fluorescence intensity of 1, i.e. the starting intensity prior to photobleaching. t = 0 s is defined as the start of fluorescence recovery. (E) Ratio of diffusivity in CVM (D_cvm_) to diffusivity in PBS (D_pbs_). (F) Unrecovered fraction of fluorescence within the time scale of measurement normalized to the initial bleached fraction. Data represent 7–9 repeated measurements per condition. * indicates a statistically significant difference (p < 0.05) compared to the same molecule in Cell-Free ROIs.

We recently showed that polyclonal HSV-binding IgG, purified from human intravenous immunoglobulin (IVIG) and added exogenously to CVM, is able to trap HSV-1 virions with comparable potency (i.e. at similar IgG concentrations) as CVM that has endogenous anti-HSV IgG [9]. Assuming comparable antigen-binding affinity between exogenous and endogenous HSV-binding IgG, this suggests that exogenous IgG molecules likely possess comparable IgG-mucin affinity as endogenous IgG. In the same study, we also showed that the rates of fluorescence recovery of exogenously added FITC-labeled IgG were only minimally slowed compared to the rates of fluorescence recovery in buffered physiological saline. The inverse ratio of the recovery rates, which reflects the extent to which diffusion of IgG in mucus was hindered relative to the same IgG in buffer, was ~0.8–0.9 where 1 indicates no affinity and 0 indicates high affinity with complete immobilization. While the observed rapid recovery is consistent with low affinity bonds between IgG and mucins in CVM, the possibility remains that endogenous IgG in CVM from a particular woman may differ from pooled blood-derived IgG in characteristics, such as IgG specificity, isotype, or heterogeneity (including glycosylation pattern), and therefore possess distinct affinity to mucins. We addressed this possibility by performing FRAP studies using CVM-derived IgG from the same donor (donor-matched IgG, or DM-IgG), prepared by pooling multiple CVM specimens from the same donor collected on consecutive days to obtain sufficient quantities of IgG for FITC labeling. We first compared the recovery kinetics of FITC-labeled IVIG to those of FITC-labeled DM-IgG in the same CVM specimens, adjusted to neutral pH to avoid significant reduction in the pH-sensitive FITC fluorescence (note that neutral pH also mimics neutralization of CVM by alkaline semen). When we performed FRAP in cell-free regions, fluorescently tagged IVIG and DM-IgG both exhibited rapid recovery of fluorescence (Fig 2A and 2C), in good agreement with our previous findings [9,12]. Interestingly, when we performed FRAP within cells, using a bleached ROI of the same dimensions, we observed substantially slower kinetics of recovery compared to in cell-free regions (Fig 2B and 2D). We further quantified FRAP measurements by calculating the inverse ratio of the recovery half-times of IgG in mucus vs. in saline (PBS), which approximates the ratio of IgG diffusivity in mucus to diffusivity in saline (D_cvm_/D_pbs_; Fig 2E), as well as the unrecovered fraction over the time scale of measurement (Fig 2F). D_cvm_/D_pbs_ averaged ~0.7–0.8 in cell-free regions for both IVIG and DM-IgG, indicative of rapid diffusion in CVM and weak affinity to mucins, but decreased significantly in cells (averages ranging from ~0.35–0.55) along with increases in the unrecovered fraction. DM-IgG exhibited a slightly (non-significantly) slower recovery rate in cell-free regions of CVM, and a greater unrecovered fraction in both cell-free and cell ROIs. Nevertheless, these observations suggest that blood-derived IVIG and CVM-derived DM-IgG generally behave similarly in CVM, with weak and transient affinity to mucins in cell-free regions of CVM. The results also suggest both types of antibody may interact more strongly with cellular constituents, resulting in markedly slower antibody diffusion within cells.

### Zenon dye added directly to CVM preferentially labels cellular bodies

**Fig 3:**
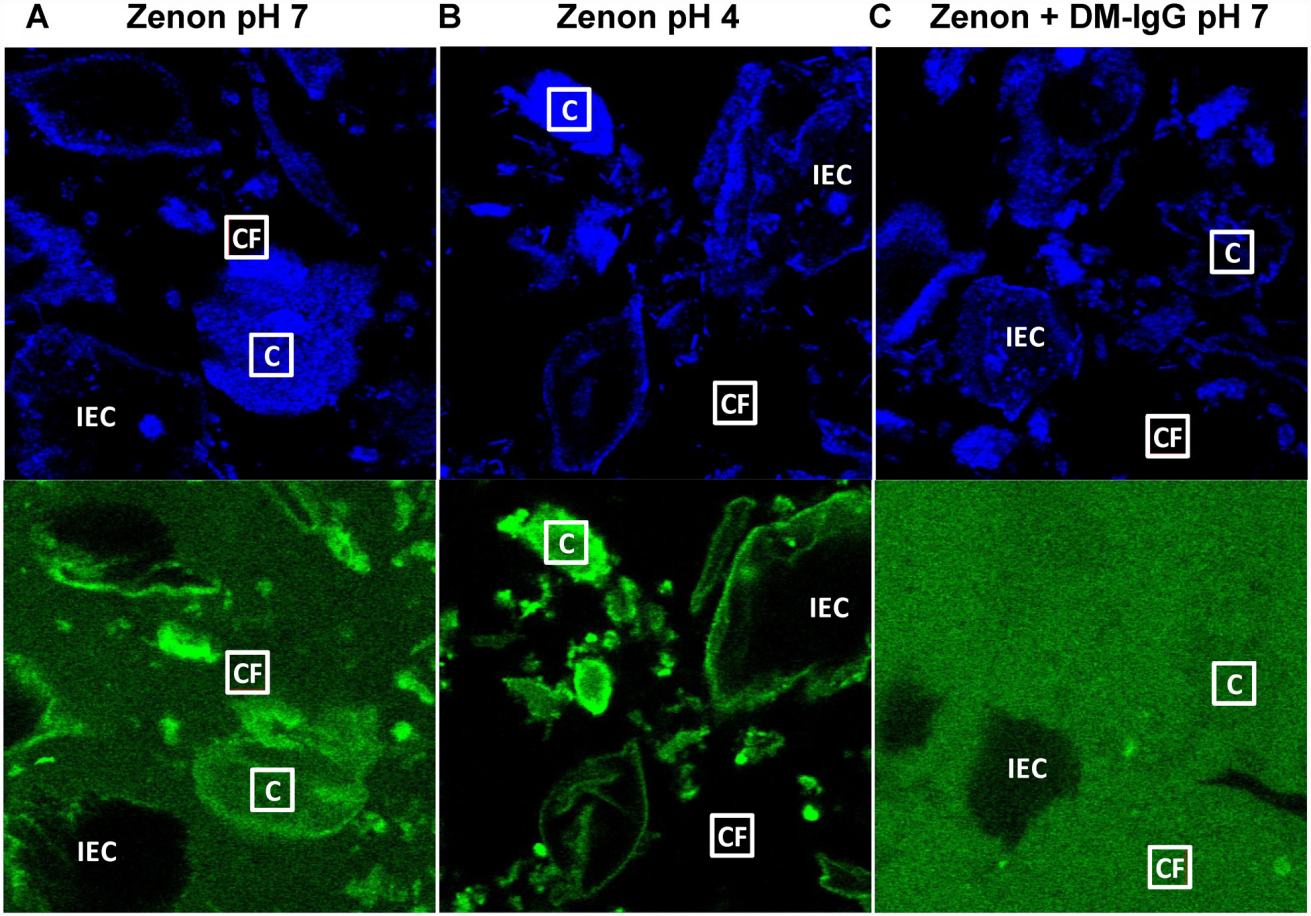
Representative blue and green channel confocal images of Zenon in human CVM. (A) Zenon added directly to pH-neutralized CVM (“Zenon pH 7”), (B) Zenon added directly to native, acidic CVM (“Zenon pH 4”), and (C) DM-IgG pre-mixed with Zenon in pH-neutralized CVM (“Zenon + DM-IgG pH 7”). Sample “Cell-Free” (“CF”) vs. “Cell” (“C”) ROIs measuring 10 μm x 10 μm are indicated, along with intact epithelial cells (“IEC”). Zenon added directly to CVM associates preferentially with cellular material, an effect that is enhanced under acidic pH, as evident by green-fluorescent cell structures against the dark background of the surrounding medium. In contrast, pre-mixed Zenon-IgG exhibits a much more uniform distribution throughout the sample, with the exception of within IEC that do not appreciably absorb IgG.

Although IVIG and DM-IgG appeared to exhibit comparably weak affinity to mucins, the results still leave open the possibility that a fraction of endogenous antibody may adhere firmly to mucins or to other mucus constituents, including shed epithelial cells. To investigate this hypothesis, we tested the use of Zenon fragments, which bind the Fc region of IgG, gently mixed into CVM to directly label endogenous antibody prior to performing FRAP measurements. Interestingly, addition of Zenon to CVM resulted in substantial spatial variations, with preferential labeling of cells and degraded cellular material compared to the surrounding mucus gel, as revealed by confocal microscopy (Fig 3A). The preferential tagging of cellular structures was even more striking in native, acidic (pH ~4) CVM, where Zenon fluorescence concentrated almost exclusively in cell bodies (Fig 3B). In contrast, addition of DM-IgG pre-mixed with Zenon to pH-neutralized CVM resulted in nearly uniform fluorescence outside of intact epithelial cells that absorbed little to no antibody (Fig 3C), in good agreement with our observations of FITC-tagged DM-IgG.

**Fig 4:**
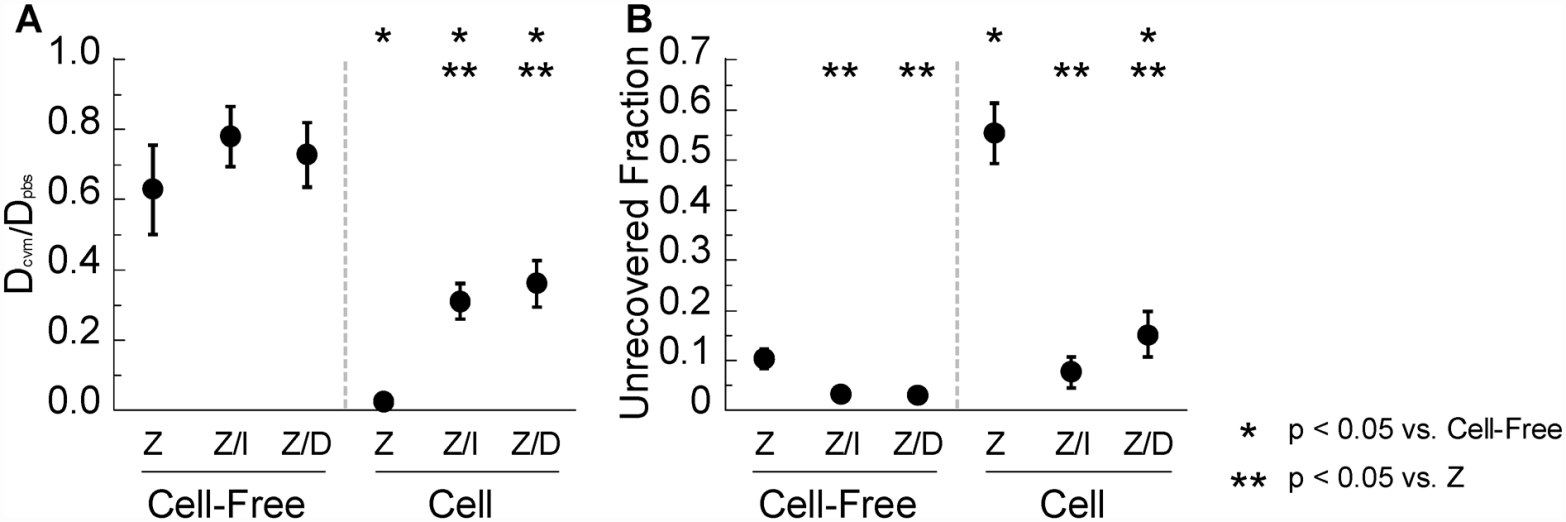
FRAP analysis of Zenon (“Z”), IVIG pre-mixed with Zenon (“Z/I”), and DM-IgG pre-mixed with Zenon (“Z/D”) in pH-neutralized human CVM. (A) Ratio of diffusivity in CVM (D_cvm_) to diffusivity in PBS (D_pbs_). (B) Unrecovered fraction of fluorescence within the time scale of measurement normalized to the initial bleached fraction. ROIs are distinguished based on whether they are in cell-free regions of the sample (“Cell-Free”) or within cells (“Cell”). Data represent 7–9 repeated measurements per condition. * indicates a statistically significant difference (p < 0.05) compared to the same molecule in Cell-Free ROIs; ** indicates a statistically significant difference (p < 0.05) compared to Zenon in ROIs of the same type.

Since spatial variations of the Zenon label may reflect differences in the affinities of free vs. bound Zenon dye with mucus constituents, we decided to perform FRAP in different regions of pH-neutralized CVM treated with either Zenon dye alone or Zenon dye pre-mixed with IgG (S3 Fig). Similar to IVIG or DM-IgG labeled with FITC, Zenon pre-mixed with IVIG and DM-IgG exhibited rapid diffusion in cell-free regions with nearly full recovered fraction (Fig 4). Interestingly, we also observed rapid diffusion and near complete recovery in cell-free regions when Zenon label was added directly into CVM. The Zenon human IgG labeling reaction occurs on the order of minutes, so the majority of the Zenon dye should be associated with IgG antibodies in CVM (whether freely diffusing or bound to mucus constituents) within the 1 hr incubation between addition of Zenon dye and FRAP measurements. Thus, if high affinity bonds are present between endogenous IgG and mucins, such mucin-bound IgG would be labeled by the Zenon dye and consequently detected in our FRAP measurements, as firmly bound IgG would fail to diffuse quickly into the bleached region from the surrounding mucus. Our observation of rapid diffusion is therefore consistent with the hypothesis that IgG molecules in cell-free portions of CVM do not form high affinity bonds with mucins. On a theoretical basis, it is also interesting to note that the number of IgG molecules in native CVM is far less than the number of mucin molecules (see S1 Text); therefore, if high affinity binding sites did exist on mucins, we would expect the vast majority of IgG to be firmly bound. We did observe a slight and non-significant reduction in the fluorescence recovery rates of endogenous IgG (directly labeled by Zenon) vs. exogenous IgG (IVIG or DM-IgG pre-mixed with Zenon) in cell-free regions of CVM. This may be attributed to a small fraction of the Zenon tag labeling IgG molecules against lactobacilli or other bacteria in CVM; nevertheless, we generally did not observe any above-background green fluorescence associated with lactobacilli, suggesting IgG bound to lactobacilli are unlikely to have any significant effect on overall FRAP measurements of IgG-mucin affinity.

Altogether, FRAP results using various IgG labeling methods, including Zenon dye directly added into CVM, consistently indicate that the majority of IgG in cell-free regions of CVM are not likely to be firmly bound to mucins, and instead exhibit only low-affinity, transient interactions with mucins or other mucus-associated factors.

**Fig 5:**
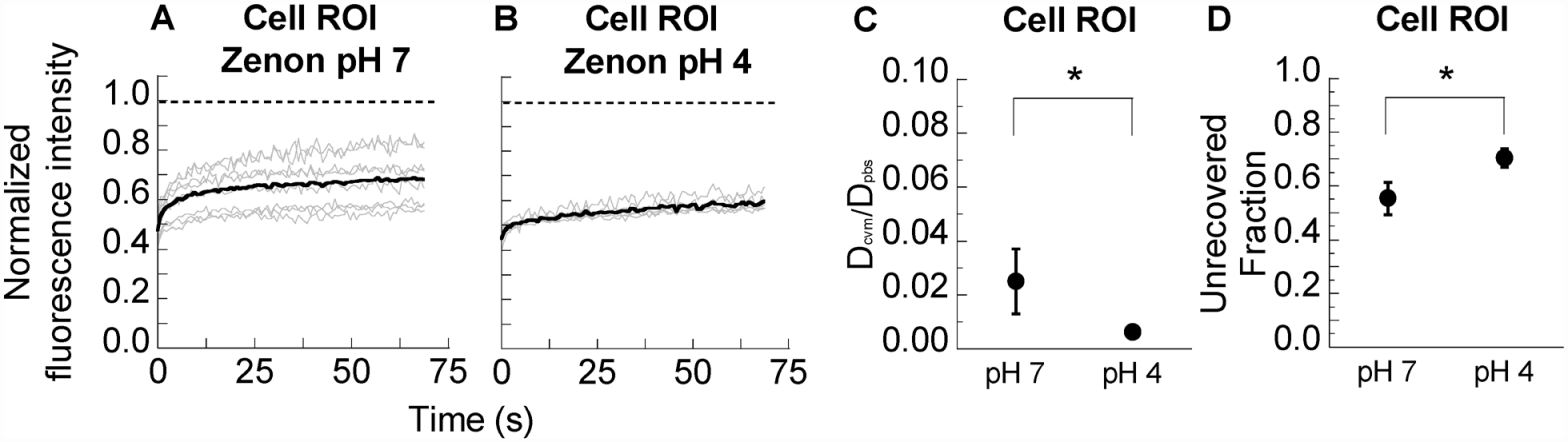
FRAP analysis of Zenon in pH-neutralized or native, acidic human CVM. (A,B) Normalized fluorescence intensities over time for photobleached ROIs of (A) Zenon in pH-neutralized human CVM (“Zenon pH 7”) and (B) Zenon in native, acidic human CVM (“Zenon pH 4”). ROIs were selected within cellular regions of the samples. Thin grey lines represent individual measurements of distinct ROIs, while thick black lines represent the average. Dashed lines represent a normalized fluorescence intensity of 1, i.e. the starting intensity prior to photobleaching. t = 0 s is defined as the start of fluorescence recovery. (C) Ratio of diffusivity in CVM (D_cvm_) to diffusivity in PBS (D_pbs_). (D) Unrecovered fraction of fluorescence within the time scale of measurement normalized to the initial bleached fraction. Data represent 7–9 repeated measurements per condition. * indicates a statistically significant difference (p < 0.05).

In ROIs within cells of pH-neutralized CVM, IVIG and DM-IgG pre-mixed with Zenon both exhibited recovery kinetics that are markedly slower than in cell-free regions, consistent with the observations in Fig 2. Interestingly, Zenon label directly mixed into pH-neutralized CVM exhibited even slower recovery kinetics and greater unrecovered fraction in cell ROIs than either IVIG or DM-IgG pre-mixed with Zenon (Fig 4). The observed slow recovery and high unrecovered fraction within cellular regions were further exacerbated with Zenon directly mixed into native CVM (pH ~4) (Fig 5). These results are consistent with the slow recovery observed in a previous FRAP study using Zenon added directly to native (i.e. presumably acidic) CVM [11]. While the observation was attributed in the prior study to high affinity bonds between IgG and secreted mucins in CVM, our results here suggest slow recovery may be specifically attributed to higher affinity interactions between IgG and cell structures.

The molecular basis substantiating the high-affinity IgG interactions with cell structures revealed by Zenon remains unclear. We observed relatively faster fluorescence recovery in cell ROI with exogenous IgG (IVIG or DM-IgG) labeled with FITC or pre-mixed with Zenon tag than with Zenon added directly to CVM. One possibility is that a substantial fraction of endogenous IgG in cell ROIs labeled by Zenon binds specifically to antigens in cellular material via Fab arms, rather than non-specifically via the Fc region. Under this scenario, the fluorescence recovery captured when performing FRAP within cells labeled by Zenon would likely include both IgG bound by Fab-antigen interactions (very slow recovery), as well as IgG that associates with cell structures via general Fc-cell interactions (relatively faster recovery). Although DM-IgG presumably possesses an IgG repertoire identical to that of endogenous IgG, it is possible that our method of isolating DM-IgG (via centrifugal separation of cells and mucins from CVM followed by affinity purification) failed to include endogenous IgG bound to cells or cellular components with higher affinity. An alternative explanation is that all IgG ‘binding’ sites on cells or cellular components may already be saturated in native CVM, and that the time necessary for exchange of endogenous bound IgG with freshly introduced DM-IgG may substantially exceed the ~1 hour of incubation time prior to our FRAP experiments. Nevertheless, assuming Zenon binds both cell-free and cell-associated IgG equally well, the addition of Zenon directly into pH-neutralized CVM likely provides an accurate representation of the true distribution of IgG in CVM, with more free, rapidly diffusing IgG in cell-free regions and more firmly bound IgG in cellular regions.

Our observations in aggregate indicate that IgG exhibits very low affinity to mucins in cell-free regions of CVM, and higher affinity to cellular structures. These results underscore the importance of the choice of fluorophore labeling method as well as the choice of bleaching regions within cells vs. cell-free regions of CVM when pursuing FRAP observations.

### Implications of IgG-mucus interactions for immune protection

More antibodies are secreted into mucus than into blood or lymph, and in the female reproductive tract, IgG is the predominant immunoglobulin in CVM secretions [9,23,24]. Nevertheless, the precise role of IgG in CVM in protecting against vaginal infections is not well understood, especially since the classical mechanisms of systemic immune protection (e.g. complement activation, opsonization, and antibody-dependent cell-mediated cytotoxicity (ADCC)) are largely absent in healthy female genital secretions, which typically have little complement activity and few if any active leukocytes [25–27]. We recently demonstrated that IgG does not need to neutralize in order to protect against infection, by showing that anti-HSV IgG at sub-neutralizing concentrations can trap HSV in murine CVM and protect mice against vaginal HSV transmission [9]. Previous studies established that individual IgG and other antibodies diffuse rapidly in mucus secretions [9,12,16], indicating IgG forms only weak and transient adhesive interactions with mucins and suggesting that individual IgG molecules would be incapable of crosslinking viruses to mucus. Nevertheless, as individual IgG molecules accumulate on a virion’s surface, the resulting array of virion-bound IgG can collectively form *multiple* weak antibody-mucin bonds between the virion and CVM, thereby generating sufficient avidity in aggregate to slow or even immobilize individual virions in mucus. This mechanism, which we have demonstrated both experimentally [9] and by computational modeling [28,29], is akin to multiple but weak links between hooks and loops on a Velcro^®^ patch that can collectively produce strong adhesion. Trapping viruses in mucus via an array of virus-bound IgG would not only reduce the flux of virus reaching target cells in the vaginal epithelium, but also enable natural mucus clearance mechanisms to eliminate trapped viruses.

While a single high affinity bond between a virion-bound antibody and a mucin fiber may also be able to trap the virion, computational modeling reveals that transient, low affinity IgG-mucin bonds are critical for ensuring efficient accumulation of IgG on the virus surface. Many viruses, including HIV and HSV, are trapped in native (acidic) CVM, but can rapidly diffuse through mucus gels under pH neutral conditions, such as in CVM exposed to semen [7–9]. Thus, antibodies must quickly diffuse through mucus to accumulate on a pathogen and trap it before it can reach the underlying cells. Moreover, we recently demonstrated that optimal virus trapping is achieved with IgG molecules that exhibit both (i) rapid antigen binding rather than very slow unbinding and (ii) relatively weak affinity with mucins [29]. Thus, contrary to common intuition, virus trapping by IgG requires neither high affinity binding to the virion nor high affinity binding to mucins. Lending further support to the notion of virus trapping via an array of individually weak IgG-mucin bonds, we also found via FRAP measurements that the affinity of a monoclonal anti-HSV antibody (HSV8 IgG) to mucins increases only slightly when the antibody is bound to HSV glycoprotein D compared to its unbound state (S4 Fig). This suggests that monomeric binding of an antigen to an IgG may not cause the Fc region to increase its affinity to mucin fibers.

Finally, the observation that IgG exhibits greater affinity within cells suggests that IgG may be absorbed (partitioned) into shed cells that have become permeable to IgG. A previous report demonstrated that the apical layers of the human vaginal epithelium contain few tight junctions and that the superficial cells appear to store IgG [30]. Preliminary observations reveal that “stored” IgG slowly diffuses back out of the cells with a half time of ~10–20 minutes, consistent with our measurements of restricted diffusion by IgG within cells or associated with cellular material. These observations suggest shed cells might potentially act as reservoirs of secreted IgG when the local environment is diluted by exogenous fluids, such as semen. Dilution of CVM by semen may create a substantial, albeit transient, reduction in the effective concentration of cell-free pathogen-binding antibody: the volume of CVM likely ranges from 1 to 2 mL in the female reproductive tract [31,32], whereas the average volume of semen is ~3 mL [33]. Thus, in the absence of reservoirs that can release additional antibody into the cell-free volume of mucus, the effective cell-free antibody concentration in mucus may be reduced locally by 3-fold or greater. Since CVM contains a high concentration of shed epithelial cells, we are currently pursuing additional measurements to quantify the magnitudes and kinetics of IgG uptake and release by these cells.

We demonstrate here how FRAP combined with careful attention to cell and cell-free regions in CVM may be used to characterize the molecular interactions between antibodies and different mucus constituents. By comparing fluorescence recovery in cell-free vs. cell ROIs, we found that antibodies form very low affinity and transient bonds with mucins in cell-free regions of CVM, but exhibit greater affinity to cells or cellular material. Low affinity bonds between antibodies and mucins may be critical to the potency with which antibodies can trap pathogens in mucus gel; thus, performing FRAP in cell-free regions of mucus may aid identification and development of antibodies that facilitate optimal pathogen trapping. Our FRAP measurements also point to a potential role of shed epithelial cells in storing and subsequently releasing the same antibodies. We expect that our work will contribute to improved understanding of the protective mechanisms of antibodies in mucus secretions, and the potential role of shed epithelial cells in serving as reservoirs of secreted antibodies.

## Materials & Methods

### Cervicovaginal mucus (CVM) collection

CVM collection was performed as published previously [1,7]. Briefly, undiluted CVM secretions, averaging 0.3 g per sample, were obtained from women of reproductive age by using a self-sampling menstrual collection device following protocols approved by the Institutional Review Board of the Johns Hopkins University (protocol # HIRB00000526). Written informed consent of participants was obtained after the nature and possible consequences of the study were explained. Participants inserted the device into the vagina for at least 30 s, removed it, and placed it into a 50 mL centrifuge tube. Samples were centrifuged at 200 xg for 2 min to collect the secretions. Samples were collected at random times throughout the menstrual cycle, and no samples were ovulatory based on visual observation (none exhibited spinnbarkeit). Samples that were non-uniform in color or consistency were discarded. Donors stated they had not used vaginal products nor participated in unprotected intercourse within 3 days prior to donating. Samples were stored at 4°C prior to use within 1 day of collection.

All samples had a native pH <4.5 (average 3.7 ± 0.2). Since fluorophores used to tag IgG for FRAP studies are pH sensitive, we titrated CVM to pH 6.8–7.1 using small volumes (~3% v/v) of 3 N NaOH, and confirmed pH using a micro pH electrode (Microelectrodes, Inc., Bedford, NH) calibrated to pH 4, 7 and 10 buffers. Unless otherwise indicated, FRAP studies were performed in pH-neutralized CVM.

### Ab purification and labeling

Human intravenous immunoglobulin (IVIG) was purchased from Privigen^®^ (≥ 98% IgG; CSL Behring, King of Prussia, PA). To obtain CVM-derived donor-matched IgG (DM-IgG), multiple CVM specimens from the same donor were collected on consecutive days and stored at -80°C until sufficient volumes were collected for IgG purification and labeling. Pooled CVM was diluted 1:5 with 1x PBS and then centrifuged for 2 min at 21,130 xg to obtain cell-free supernatant containing antibody. Total IgG was purified from antibody supernatant using Pierce^®^ Protein A/G agarose (product #20422; Thermo Scientific, Rockford, IL). Elution fractions were buffer exchanged into Dulbecco’s Phosphate Buffer Saline (DPBS, product #14190–144; Thermo Scientific, Rockford, IL) using a 50 kDa molecular weight cutoff (MWCO) concentrator (Spin-X UF 20 mL, product #431490; Corning, Corning, NY). Concentrated DM-IgG was sterilized by centrifugation through a 0.22 μm filter (Costar 8160; Corning, Corning, NY). This method yields IgG concentrations ranging from ~0.1–2 mg/mL [9], in good agreement with previously published values [24] and suggesting the majority of IgG in mucus is not firmly bound to mucins or other mucus components. HSV8 anti-gD monoclonal IgG was produced in HEK 293 cells as previously described [9].

Fluorescein isothiocyanate (FITC; Life Sciences, Grand Island, NY) was used to label IVIG, DM-IgG and HSV8 IgG following manufacturer protocol. Labeled antibodies were purified using a Protein A/G column (0.2 mL resin; Thermo Scientific, Rockford, IL). The column was washed with excess PBS to remove unconjugated FITC. Bound Ab was then eluted using IgG Elution Buffer (Thermo Scientific, Rockford, IL); each elution consisted of three 3 mL volumes of elution buffer, and was collected into tubes containing 100 μL of 10 mM sodium phosphate pH 6 to neutralize the elutions. Eluted FITC-labeled IgG was concentrated using a 50 kDa MWCO concentrator (Corning 431490), supplemented with sodium azide (final concentration 0.03%) and stored at 4°C until use.

IgG concentration was determined using the Human Isotyping Kit (HGAMMAG-301K; Millipore, Billerica, MA) according to manufacturer protocol. Briefly, 20X stock isotyping beads were vortexed, sonicated, diluted to 1X, and incubated with 50 μL of serially diluted IgG solution at 1:2 beads:IgG volume ratio. After 1 hr, the beads were separated using a magnetic plate, and washed twice with wash buffer. The beads were then incubated with 25 μL of 1X anti-Human Kappa and Lambda-PE for 1 hr, washed twice, and resuspended in Luminex Drive fluid. Fluorescence intensities indicative of immunoglobulin levels were measured using the Luminex MAGPIX system, and data analysis was performed using Milliplex Analyst (v3.5.5.0; Vigene Tech Inc., Carlisle, MA). All incubations were carried out at room temperature in the dark with vigorous agitation.

### Fluorescence recovery after photobleaching (FRAP) in CVM

FRAP measurements were performed for the following antibodies and/or labels: (1) IVIG; (2) DM-IgG; (3) Zenon Fab fragments (Zenon Alexa Fluor^®^ 488 Human IgG Labeling Kit; Life Sciences, Grand Island, NY); (4) Zenon pre-mixed with IVIG; (5) Zenon pre-mixed with DM-IgG; (6) HSV8 IgG; and (7) HSV8 IgG pre-mixed with HSV1 gD glycoprotein (H2033-19F; US Biological, Salem, MA). Zenon was mixed into CVM at an approximately 1:1 molar ratio with endogenous IgG. According to manufacturer protocol, at least 3 molecules of Zenon can label each IgG molecule; thus, excess free Zenon is unlikely. We also could not appreciably photobleach Zenon in saline solution, since the molecules (~1/3 the size of IgG) diffuse back into the photobleached region too quickly to capture at the frame rate we used. This suggests any contribution from free Zenon molecules to FRAP recovery curves would be negligible.

All pre-mix conditions were incubated for 1 hr at room temperature prior to addition to CVM. Antibody and/or Zenon solutions (final concentrations in CVM of 0.1–1.5 mg/mL) were added to 10 μL of CVM placed in a custom-made glass chamber, gently stirred to achieve uniform distribution, and incubated for 1 hr at 37°C prior to microscopy. PBS was added as needed to ensure the same degree of CVM dilution (~20% v/v) across all conditions; this degree of dilution does not result in bulk visual differences in the mucus. PBS controls were similarly prepared and imaged. Based on experiments with washed vaginal epithelial cells, we have observed that the cells absorb IgG with a half-time of 10–20 min. Thus, the 1 hr incubation provides sufficient time for cells to absorb most of the IgG they are capable of absorbing prior to FRAP measurements.

FRAP experiments were conducted using a Zeiss LSM 510 META Confocal microscope with a 40x objective (numerical aperture 1.3). Cells and cellular bodies were identified based on morphology and auto-fluorescence under excitation by a 405 nm laser. Square ROIs (approximately 10 μm x 10 μm) were selected in cell-free or cellular regions. For cell ROI, we first performed a Z-stack and adjusted the Z-position to ensure ROI were within the cell volume rather than on the cell surface. We also required that selected cells had sufficient fluorescence for reliable FRAP measurement. Because the shed cells exhibit varying stages of degradation, the degree to which their membranes are permeabilized and thus how much IgG the cells absorb also vary, with some cells appearing completely dark (cells with intact membranes) while others exhibit fluorescence intensity comparable to or exceeding that of the surrounding mucus.

For both cell-free and cell ROI, we repeated FRAP experiments 7–9 times for each condition (i.e., antibody or labeling method). ROIs were bleached with 405/488 lasers at 100% laser intensity over a duration of ~4.5 s. After bleaching, fluorescence recovery was monitored over ~70 s with frames acquired every ~0.8 s. Fluorescence intensity for ROIs was calculated using ZEN Imaging Software (Carl Zeiss, Thornwood, NY), and values were normalized to the starting intensity and adjusted for photobleaching of the entire image over time based on control ROIs. The half time to full fluorescence recovery was estimated from the resulting recovery curves or projected based on the slope of the curve for cases where half recovery was not reached over the time course of measurement. The inverse ratio of recovery half-times in PBS vs. CVM was calculated to approximate the ratio of IgG diffusivity in mucus to diffusivity in saline (D_cvm_/D_pbs_). The unrecovered fraction was defined as the approximate fraction of the starting fluorescence intensity yet to be recovered at the end of the measurement, normalized to the bleached fraction.

### Statistical analysis

Statistical comparisons were limited to two groups. A two-tailed Student’s t-test with unequal variance was used for all comparisons. Differences were deemed significant at an alpha level of 0.05. All values are reported as mean ± SEM unless otherwise indicated.

## Supporting Information

S1 Fig Representative (A) blue, (B) green, (C) DIC and (D) composite channel confocal image of human CVM, with no FITC-labeled antibody added. (PDF)

S2 Fig Representative confocal image of washed vaginal epithelial cells incubated in FITC-IgG solution over 3 hours, illustrating antibody absorption (partitioning) into the cells yielding substantially brighter intensity than the surrounding solution. (PDF)

S3 Fig Normalized fluorescence intensities over time for photobleached ROIs of (A,B) Zenon, (C,D) IVIG pre-mixed with Zenon (Zenon + IVIG), and (E,F) DM-IgG pre-mixed with Zenon (Zenon + DM-IgG) in pH-neutralized human CVM. ROIs are distinguished based on whether they are (A,C,E) in cell-free regions of the sample (“Cell-Free ROI”) or (B,D,F) within cells (“Cell ROI”). Thin grey lines represent individual measurements of distinct ROIs, while thick black lines represent the average. Dashed lines represent a normalized fluorescence intensity of 1, i.e. the starting intensity prior to photobleaching. t = 0 s is defined as the start of fluorescence recovery.
(PDF)

S4 Fig (A,B) Normalized fluorescence intensities over time for photobleached ROIs of FITC-labeled (A) HSV8 IgG (“HSV8 -gD") and (B) HSV8 premixed with HSV-1 gD glycoprotein (“HSV8 +gD”) in pH-neutralized human CVM. ROIs were selected in cell-free regions of the samples. Thin grey lines represent individual measurements of distinct ROIs, while thick black lines represent the average. Dashed lines represent a normalized fluorescence intensity of 1, i.e. the starting intensity prior to photobleaching. t = 0 s is defined as the start of fluorescence recovery. (C) Ratio of diffusivity in CVM (D_cvm_) to diffusivity in PBS (D_pbs_). (D) Unrecovered fraction of fluorescence within the time scale of measurement normalized to the initial bleached fraction.
(PDF)

S1 Text Concentration of IgG vs. mucin molecules in human CVM. (PDF)
