# Supplementary material for: Diffusion of Immunoglobulin G in Shed Vaginal Epithelial Cells and in Cell-Free Regions of Human Cervicovaginal Mucus
Source: PLoS One. 2016 Jun 30;11(6):e0158338. doi: 10.1371/journal.pone.0158338 (PMC4928780; doi:10.1371/journal.pone.0158338)
Supplement: S1 Fig — (PDF) [file pone.0158338.s001.pdf]

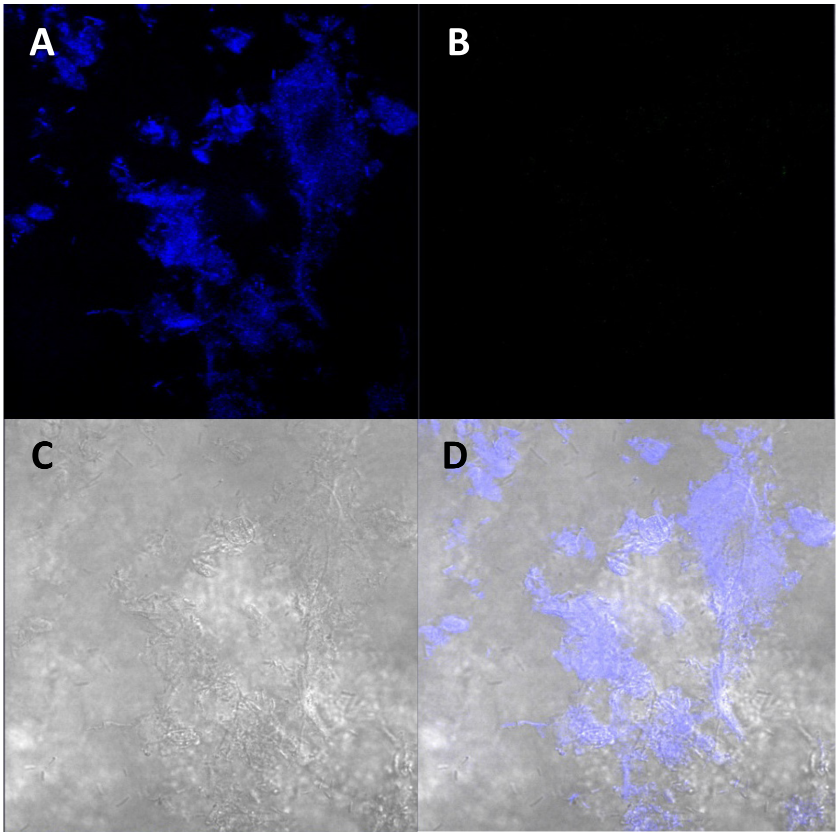

S1 Fig. Representative (A) blue, (B) green, (C) DIC and (D) composite channel confocal image of human CVM, with no FITC-labeled antibody added.
