# Supplementary material for: Diffusion of Immunoglobulin G in Shed Vaginal Epithelial Cells and in Cell-Free Regions of Human Cervicovaginal Mucus
Source: PLoS One. 2016 Jun 30;11(6):e0158338. doi: 10.1371/journal.pone.0158338 (PMC4928780; doi:10.1371/journal.pone.0158338)
Supplement: S2 Fig — (PDF) [file pone.0158338.s002.pdf]

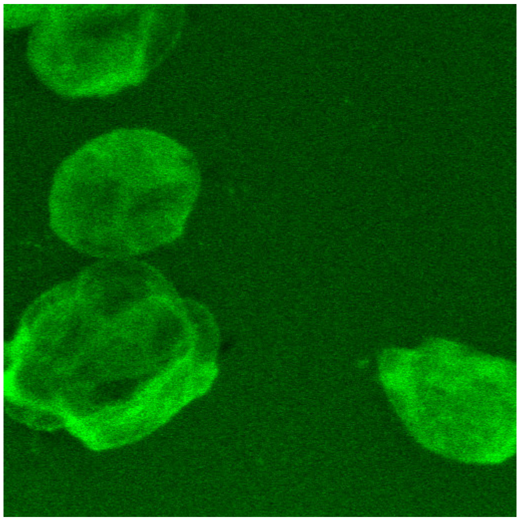

S2 Fig. Representative confocal image of washed vaginal epithelial cells incubated in FITC-IgG solution over 3 hours, illustrating antibody absorption (partitioning) into the cells yielding substantially brighter intensity than the surrounding solution.
