# Supplementary material for: Diffusion of Immunoglobulin G in Shed Vaginal Epithelial Cells and in Cell-Free Regions of Human Cervicovaginal Mucus
Source: PLoS One. 2016 Jun 30;11(6):e0158338. doi: 10.1371/journal.pone.0158338 (PMC4928780; doi:10.1371/journal.pone.0158338)
Supplement: S3 Fig — ROIs are distinguished based on whether they are (A,C,E) in cell-free regions of the sample (“Cell-Free ROI”) or (B,D,F) within cells (“Cell ROI”). Thin grey lines represent individual measurements of distinct ROIs, while thick black lines represent the average. Dashed lines represent a normalized fluorescence intensity of 1, i.e. the starting intensity prior to photobleaching. t = 0 s is defined as the start of fluorescence recovery. (PDF) [file pone.0158338.s003.pdf]

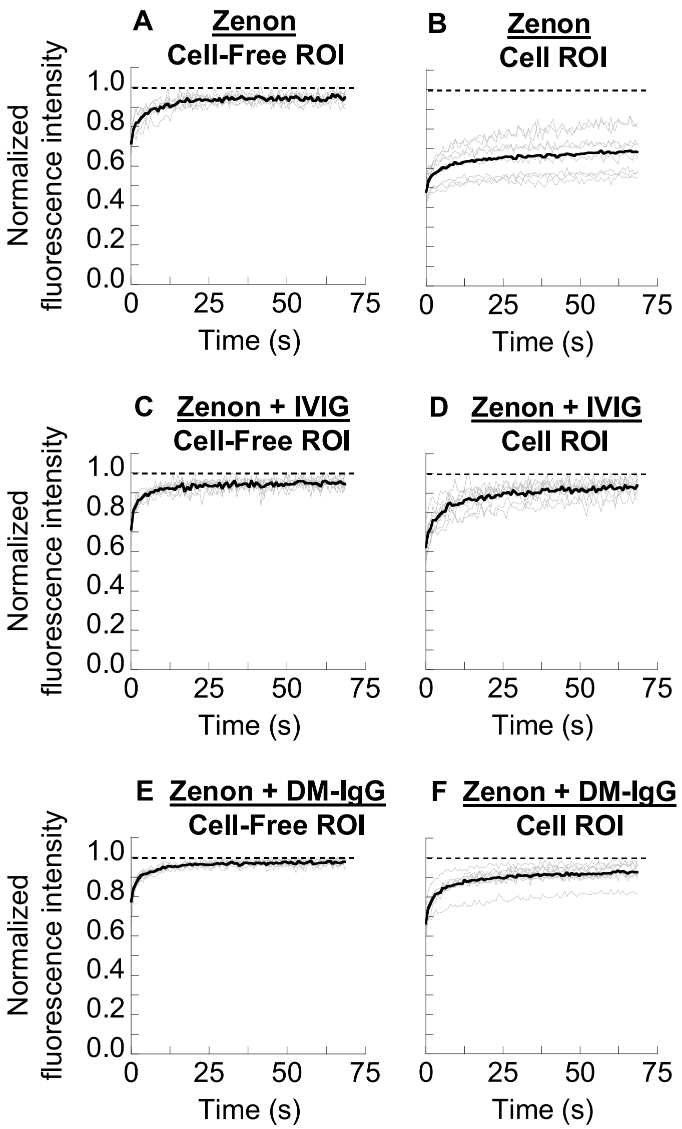

S3 Fig. Normalized fluorescence intensities over time for photobleached ROIs of (A,B) Zenon, (C,D) IVIG pre-mixed with Zenon (Zenon + IVIG), and (E,F) DM-IgG pre-mixed with Zenon (Zenon + DM-IgG) in pH-neutralized human CVM. ROIs are distinguished based on whether they are (A,C,E) in cell-free regions of the sample (“Cell-Free ROI”) or (B,D,F) within cells (“Cell ROI”). Thin grey lines represent individual measurements of distinct ROIs, while thick black lines represent the average. Dashed lines represent a normalized fluorescence intensity of 1, i.e. the starting intensity prior to photobleaching. t = 0 s is defined as the start of fluorescence recovery.
