# Supplementary material for: Diffusion of Immunoglobulin G in Shed Vaginal Epithelial Cells and in Cell-Free Regions of Human Cervicovaginal Mucus
Source: PLoS One. 2016 Jun 30;11(6):e0158338. doi: 10.1371/journal.pone.0158338 (PMC4928780; doi:10.1371/journal.pone.0158338)
Supplement: S4 Fig — (A,B) Normalized fluorescence intensities over time for photobleached ROIs of FITC-labeled (A) HSV8 IgG (“HSV8 -gD") and (B) HSV8 premixed with HSV-1 gD glycoprotein (“HSV8 +gD”) in pH-neutralized human CVM. ROIs were selected in cell-free regions of the samples. Thin grey lines represent individual measurements of distinct ROIs, while thick black lines represent the average. Dashed lines represent a normalized fluorescence intensity of 1, i.e. the starting intensity prior to photobleaching. t = 0 s is defined as the start of fluorescence recovery. (C) Ratio of diffusivity in CVM (Dcvm) to diffusivity in PBS (Dpbs). (D) Unrecovered fraction of fluorescence within the time scale of measurement normalized to the initial bleached fraction. (PDF) [file pone.0158338.s004.pdf]

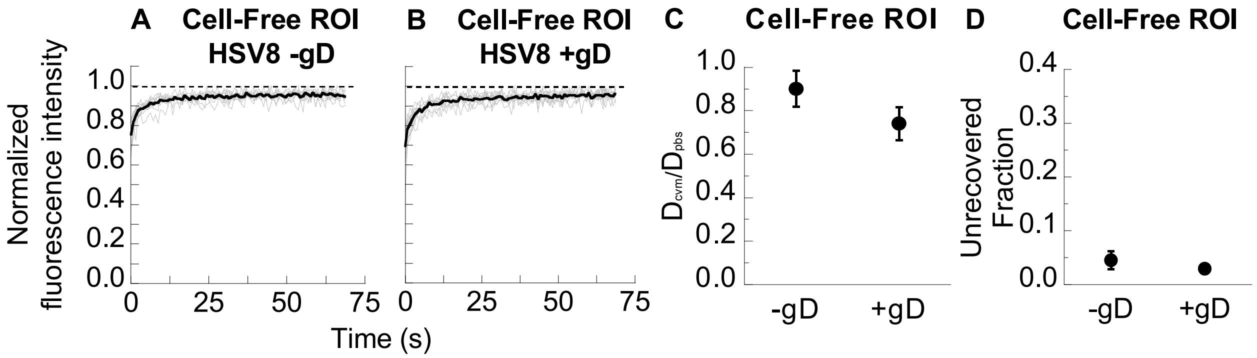

S4 Fig. (A,B) Normalized fluorescence intensities over time for photobleached ROIs of FITC-labeled (A) HSV8 IgG ("HSV8 -gD") and (B) HSV8 premixed with HSV-1 gD glycoprotein ("HSV8 +gD") in pH-neutralized human CVM. ROIs were selected in cell-free regions of the samples. Thin grey lines represent individual measurements of distinct ROIs, while thick black lines represent the average. Dashed lines represent a normalized fluorescence intensity of 1, i.e. the starting intensity prior to photobleaching. t = 0 s is defined as the start of fluorescence recovery. (C) Ratio of diffusivity in CVM (D_{cvm}) to diffusivity in PBS (D_{pbs}). (D) Unrecovered fraction of fluorescence within the time scale of measurement normalized to the initial bleached fraction.
