# Supplementary material for: Diffusion of Immunoglobulin G in Shed Vaginal Epithelial Cells and in Cell-Free Regions of Human Cervicovaginal Mucus
Source: PLoS One. 2016 Jun 30;11(6):e0158338. doi: 10.1371/journal.pone.0158338 (PMC4928780; doi:10.1371/journal.pone.0158338)
Supplement: S1 Text — (PDF) [file pone.0158338.s005.pdf]

S1 Text. Concentration of IgG vs. mucin molecules in human CVM

Assuming 2.5% mucin content by weight at an average monomer MW of 500 kDa [1-3], the concentration of individual mucin molecules in CVM is approximately 50 μM. This substantially exceeds the concentration of endogenous IgG, estimated to be ~6.7 μM IgG at ~1 mg/mL [4]. We make an entirely hypothetical postulate: if there are high affinity IgG-binding sites on mucins, there should be at least one such site accessible on each mucin molecule. Thus, even with the addition of exogenous IgG in our FRAP studies, the concentration of total IgG in CVM in our experiments was still well below potentially saturating levels. This would suggest that, if high affinity bonds did exist between IgG and mucins, the majority of IgG molecules should be firmly bound to mucins, and unable to diffuse quickly into a bleached ROI. Since our FRAP experiments demonstrated rapid IgG diffusion in cell-free regions of CVM, irrespective of IgG type or labeling method, our results strongly suggest the vast majority of IgG molecules are freely diffusing, and there are few if any high affinity binding sites for IgG on mucins.

1. Andersch-Bjorkman Y, Thomsson KA, Holmen Larsson JM, Ekerhovd E, Hansson GC (2007) Large scale identification of proteins, mucins, and their O-glycosylation in the endocervical mucus during the menstrual cycle. Mol Cell Proteomics 6: 708-716.
2. Lai SK, Wang YY, Wirtz D, Hanes J (2009) Micro- and macrorheology of mucus. Adv Drug Deliv Rev 61: 86-100.
3. Raynal BD, Hardingham TE, Thornton DJ, Sheehan JK (2002) Concentrated solutions of salivary MUC5B mucin do not replicate the gel-forming properties of saliva. Biochem J 362: 289-296.
4. Usala SJ, Usala FO, Haciski R, Holt JA, Schumacher GF (1989) IgG and IgA content of vaginal fluid during the menstrual cycle. J Reprod Med 34: 292-294.
